# T-2 Toxin Exposure Induces Apoptosis in TM3 Cells by Inhibiting Mammalian Target of Rapamycin/Serine/Threonine Protein Kinase(mTORC2/AKT) to Promote Ca^2+^Production

**DOI:** 10.3390/ijms19113360

**Published:** 2018-10-27

**Authors:** Ji Wang, Chenglin Yang, Zhihang Yuan, Jine Yi, Jing Wu

**Affiliations:** 1 College of Veterinary Medicine, Hunan Agricultural University, Changsha 410128, China; wangjics@163.com (J.W.); yang4698@163.com (C.Y.); s51857176@gmail.com (Z.Y.); yibinzhen@163.com (J.Y.); 2 Hunan Collaborative Innovation Center of Animal Production Safety, Changsha 410128, China; 3 Hunan Engineering Research Center of Veterinary Drug, Hunan Agricultural University, Changsha 410128, China

**Keywords:** T-2 toxin, apoptosis, TM3 cell, mTOR, AKT, calcium

## Abstract

Although mTOR (the mammalian target of rapamycin) can regulate intracellular free Ca^2+^concentration in normal cultured podocytes, it remains elusive as to how mTORC2/AKT-mediated Ca^2+^participates in the process of T-2 toxin-induced apoptosis. The potential signaling responsible for intracellular Ca^2+^ concentration changes was investigated using immunoblot assays in an in vitro model of TM3 cell injury induced by T-2 toxin. Changes in Ca^2+^ were assessed using the Ca^2+^-sensitive fluorescent indictor dye Fura 2-AM. The cytotoxicity of TM3 cells was assessed with an MTT bioassay, and apoptosis was measured using Annexin V-FITC staining. Following T-2 toxin treatment, the growth of cells, phospho-mTORSer2481, phospho-mTORSer2448, and phospho-AktSer473 were significantly decreased in a time-dependent manner, whereas Ca^2+^ and apoptosis were increased. T-2 toxin-induced apoptosis was prevented by BAPTA-AM (a Ca^2+^chelator) and MHY1485 (an mTOR activator), and the application of mTOR activator MHY1485 also prevented the increase of intracellular free Ca^2+^concentration in TM3 cells. Our results strongly suggest that T-2 toxin exposure induces apoptosis in TM3 cells by inhibiting mTORC2/AKT to promote Ca^2+^ production.

## 1. Introduction

Mycotoxins are a very numerous and diverse group of secondary metabolites of molds that cause toxicological effects in mammals [1]. It is estimated that before or after harvest, approximately 25% of the world’s agricultural commodities are contaminated to some extent with mycotoxins [2]. One of the most notorious mycotoxins that present a potential hazard to human and animal health is T-2 toxin. This toxin is a structural derivative of the trichothecene ring system; trichothecenes are synthetized by several *Fusarium* species, such as *F. sporotrichioides*, *F. langsethiae*, *F. acuminatum*, and *F. poae* [3]. T-2 toxin is the most toxic member of the trichothecene family; the toxin primarily exerts effects that are similar to those of a radiation injury by negatively impacting protein levels and RNA and DNA synthesis in eukaryotic cells, thus inhibiting cellular functions, such as the cell cycle and resulting in apoptosis [2,4,5]. Oral, parenteral, and cutaneous exposure to T-2 toxin manifests deleterious effects in some experimental animal modes, which exhibit apoptosis in various tissues and organs, including the skin, kidney, brain, hematopoietic, lymphoid, gastrointestinal bone marrow, and reproductive organs [6,7,8,9]. In light of the great harm to the health of humans and livestock, the toxicological effects of T-2 toxin were reported in the Joint Food and Agriculture Organization/ World Health Organization (FAO/WHO) Expert Committee on Food Additives [10].

T-2 toxin-induced apoptosis has been considered to be one of the important mechanisms of its toxic effects. T-2 toxin has been documented to induce apoptosis in various cell types, such as human chondrocytes, HL-60, Hela, Bel-7402, U937 cells, Vero, and human liver cells in vitro; this involves Fas, p53, Bcl-xL, Bcl-2, Bax caspase-9, and caspase-3 signaling pathways [11,12,13]. In addition, several studies have demonstrated that excessive intracellular calcium concentration, one of the most important second messengers in multiple cellular activities, subsequently leads to the depolarization of mitochondria and apoptosis [14,15]. The Ca^2+^ induced by T-2 toxin appears to be involved in the activation of several caspases, resulting in apoptosis [16].

Mammalian/mechanistic target of rapamycin (mTOR), a serine/threonine protein kinase (AKT), plays a crucial role in cell growth, proliferation, and apoptosis [17,18]. Recently, it was reported that mTOR could regulate intracellular Ca^2+^ in cultured normal podocytes [19,20]. Hence, in this study, we focused on the distinct role of mTOR signaling and investigated how Ca^2+^ contributes to T-2 toxin-induced TM3 cell apoptosis.

## 2. Results

### 2.1. TM3 Cell Viability

**Figure 1:**
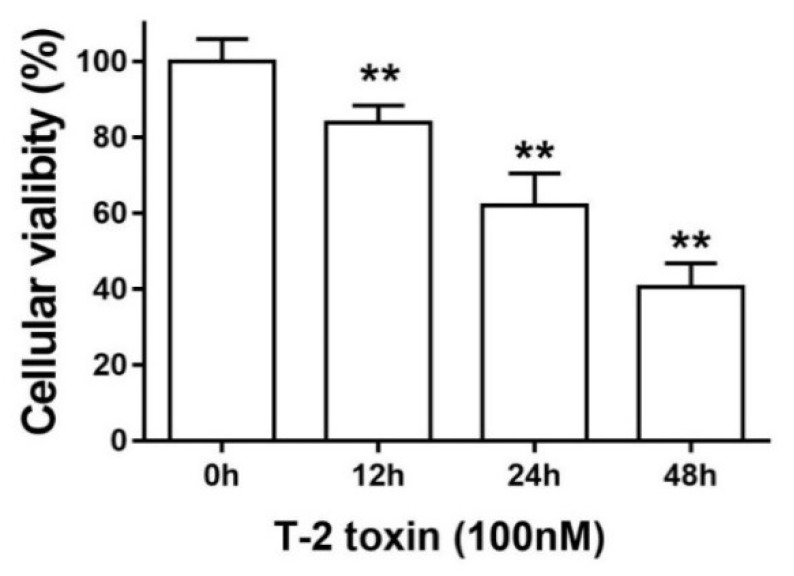
T-2 toxin decreases viability in TM3 cells. ** indicates *p* < 0.01 when compared with the untreated group. Each experiment was performed and repeated at least three times.

An MTT assay was used to measure the viability of TM3 cells after treatment with T-2 toxin in different times. As shown in Figure 1, the cells’ viability was influenced by T-2 toxin in a time-dependent manner. Thus, the results suggest that 12 h exposure to T-2 toxin at the concentrate of 100 nM significantly (*p* < 0.01) reduced the TM3 cell viability.

### 2.2. T-2 Toxin Induces Intrinsic Apoptosis in TM3 Cells

**Figure 2:**
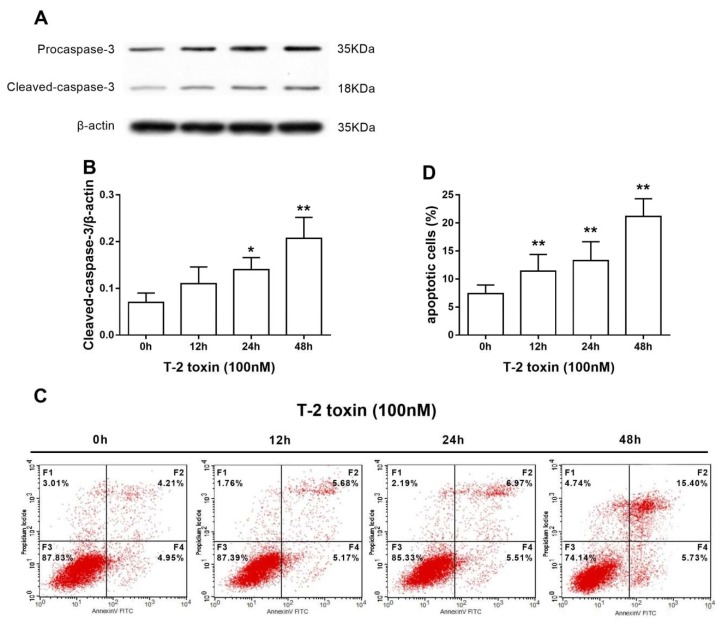
T-2 toxin induces intrinsic apoptosis in TM3 cells. (**A**) Expression of cleaved-caspase-3 was analyzed by Western blotting. (**B**) Level of cleaved-caspase-3 was quantified by densitometry. (**C**) Apoptosis was analyzed by Annexin V/PI assays in TM3 cells. (**D**) Percentage of apoptotic cells treated by T-2 toxin. T-2 toxin induced apoptosis in a time-dependent manner. * indicates *p* < 0.05 and ** indicates *p* < 0.01 when compared with the untreated group. Each experiment was performed and repeated at least three times.

Caspase-3 is the key executioner in apoptosis [21], and activated caspase-3 is cleaved into various proteins, which kill cells via apoptosis. Figure 2A,B shows that significantly higher levels of cleaved caspase-3 were found in TM3 cells that were treated with T-2 toxin for 24 and 48 h. In addition, flow cytometry using Annexin-V and PI was performed to determine whether T-2 toxin induced apoptosis. As shown in Figure 2C,D, the percentage of apoptotic cells increased 12–48 h after T-2 toxin treatment in TM3 cells. Collectively, these data confirm that T-2 toxin induced TM3 cells apoptosis in a time-dependent manner.

### 2.3. Induction of Ca^2+^Is Involved in T-2 Toxin-Mediated TM3 Cells Apoptosis

**Figure 3:**
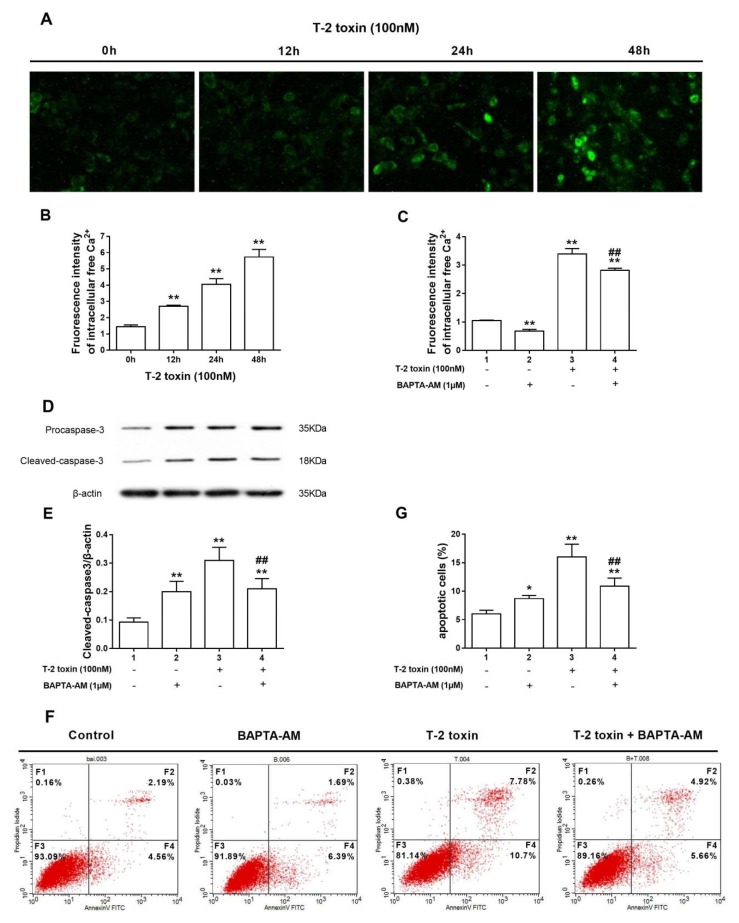
Induction of Ca^2+^ is involved in T-2 toxin-mediated TM3 cell apoptosis. (**A**) Representative images of the fluorescence intensity of intracellular free Ca^2+^ measured with Fura-2/AM, images were captured at 200× magnification. (**B**) T-2 toxin induced an increase in the free Ca^2+^ intracellular concentration in a time-dependent manner. (**C**) BAPTA-AM pretreatment attenuated the increase of free Ca^2+^ intracellular concentration. (**D**) Expression of cleaved-caspase-3 was analyzed by Western blot assay. (**E**) Level of cleaved-caspase-3 was quantified by densitometry. (**F**) Apoptosis was analyzed by Annexin V/PI assays in TM3 cells. (**G**) Percentage of apoptotic cells treated with T-2 toxin and (or) BAPTA-AM. BAPTA-AM attenuated the increase of percentage of apoptotic cells caused by treatment with T-2 toxin. * indicates *p* < 0.05 and ** indicates *p* < 0.01 when compared with the control group. ## indicates *p* < 0.01 when the co-treated group is compared with the group only treatment with T-2 toxin. Each experiment was performed and repeated at least three times.

The Ca^2+^-sensitive dye Fura2-AM was used to determine the relationship between the Ca^2+^ signal and apoptosis induced by the T-2 toxin. The results that are shown in Figure 3A,B demonstrate that after incubation with T-2 toxin, the intracellular Ca^2+^concentration was time-dependently enhanced. BAPTA-AM was used in our experiment to verify whether the Ca^2+^was involved in T-2 toxin-mediated apoptosis. When compared with the control group, BAPTA-AM treatment showed a significant decline in the Ca^2+^ (Figure 3C) and remarkably increased expression of cleaved caspase-3 (*p* < 0.05), consequently inducing apoptosis (Figure 3D–G). Notably, T-2 toxin-mediated elevation of the cleaved-caspase-3 and apoptosis was diminished by co-treatment of T-2 toxin with BAPTA-AM (Figure 3D–G).

### 2.4. The mTOR/Akt Pathway Inhibition Is Related to T-2 Toxin-Mediated Apoptosis

**Figure 4:**
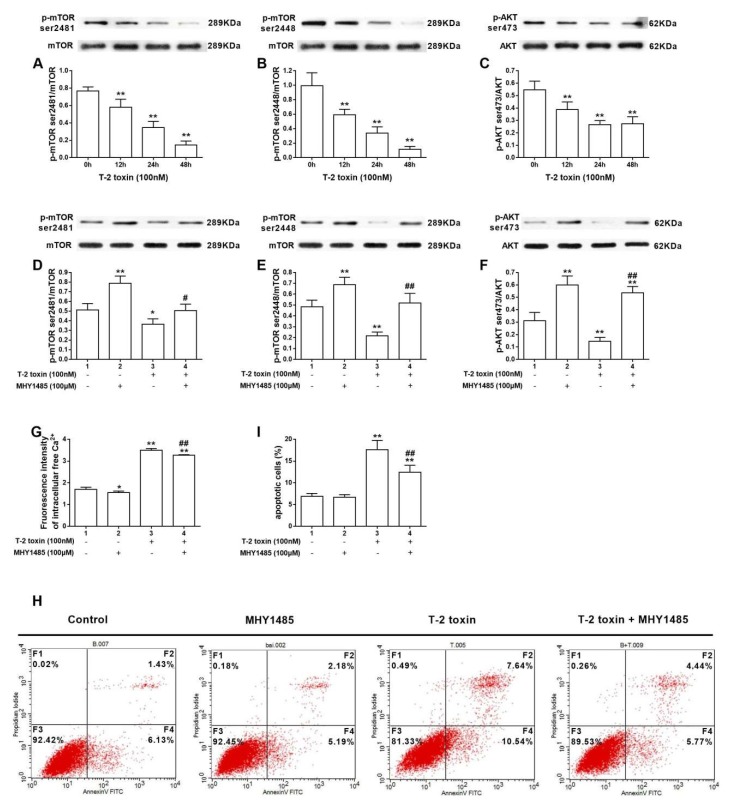
Mammalian Target of Rapamycin/Serine/Threonine Protein Kinase (mTOR/Akt) pathway inhibition is related to T-2 toxin-mediated apoptosis. T-2 toxin reduced the relative expression of p-mTOR ser 2481 (**A**), p-mTOR ser 2448 (**B**), and p-AKT ser 473 (**C**). MHY1485 attenuated the T-2 toxin-caused suppression of phosphorylation of mTOR at ser 2481 (**D**), ser 2448 (**E**), and AKT at ser 473 (**F**). MHY1485 inhibited free Ca^2+^ intracellular concentration (**G**) and protected TM3 cells from apoptosis (**H**,**I**). * indicates *p* < 0.05 and ** indicates *p* < 0.01 when compared with the control group. ## indicated *p* < 0.01 when the co-treated group is compared with the group only treatment with T-2 toxin. Each experiment was performed and repeated at least three times.

mTOR(the mammalian target of rapamycin) is a serine/threonine kinase that is regulated by phosphoinositide 3-kinase(PI3K)/Akt signaling. mTOR complexes are categorized into two multi-protein complexes, which are mTOR complex 1 (mTORC1) and mTOR complex 2 (mTORC2). mTORC1 activation is characterized predominantly by phosphorylation at Ser 2448, while mTORC2 activation is characterized predominantly by phosphorylation at Ser 2481, resulting in the phosphorylation of Akt/Protein Kinase B at Ser 473 [20,22]. As illustrated in Figure 4A, the phosphorylation of mTOR at Ser2481 decreased significantly in a time-dependent manner in T-2 toxin-treated TM3 cells. Similarly, the phosphorylation of mTOR at Ser 2448 showed the same trend (Figure 4B). Consistently, we detected a time-dependent suppression of phospho-Akt Ser 473 (Figure 4C). MHY1485, a novel small molecular activator of mTOR [23], was used to prove whether the mTOR/Akt signaling pathway was involved in the intracellular Ca^2+^ concentration that was induced by T-2 toxin. Significantly, the inhibitory effect of T-2 toxin on mTOR/Akt was dramatically attenuated by MHY1485 (Figure 4D–F). Meanwhile, MHY1485 significantly recovered T-2 toxin-mediated enhancement of Ca^2+^, and protected TM3 cells from apoptosis induced by T-2 toxin (Figure 4G–I).

## 3. Discussion

TM3 cells are the main source of androgen for spermatogenesis. For males, the maturation of sexual organs and the emergence of sexual characteristics are directly influenced by TM3 cells. Yang’s experiment has demonstrated that T-2 toxin has negative effects on male fertility and other reproductive pathologies [24]. More research has proven that TM3 cells are influenced by T-2 toxin in a dose-dependent manner and has revealed the mechanisms of cytotoxicity [25,26,27,28]. Our previous studies found that T-2 toxin decreased antioxidant scavenging enzymes, increased lipid peroxidation and ROS production, and contributed to TM3 cells apoptosis. Moreover, activating the ROS-mediated mitochondrial pathway is the mechanism of T-2 toxin-induce apoptosis [29].

To investigate the temporal relationship between T-2 toxin and TM3 cells, the cytotoxicity of T-2 toxin was determined at different times. We observed that T-2 toxin inhibited cellular viability in a time-dependent manner. When compared with the control group, T-2 toxin significantly increased the expression of cleaved caspase-3, a crucial factor in apoptosis. T-2 toxin strongly increased the percentage of apoptotic cells at 12 h. Zhuang et al. reported that T-2 toxin was linked with oxidative stress and the mitochondrial pathway in a time-dependent manner in Hela, Bel-7402, and Chang liver cells [13]. Similarly, in the current study, the results that are presented above clearly show that T-2 toxin induces intensive cytotoxicity in TM3 Leydig cells.

As an important second messenger in cells, Ca^2+^ is closely related to a series of important physiological and pathological processes, such as proliferation, differentiation, development, and cell death. The accumulated evidence has demonstrated that when cells are stimulated with T-2 toxin, it leads to the activation of store-operated calcium entry, which plays a crucial role in regulating Ca^2+^ signaling and cellular responses in arterial smooth muscle cells [30]. Notably, Decuypere et al has reported that Ca^2+^ is the key player in the canonical mTOR-controlled autophagy pathway [31]. But, there was few research about relationship of T-2 toxin-induces apoptosis and Ca^2+^ signal way, we first discuss the role of Ca^2+^ in T-2 toxin-mediated apoptosis. In our experiment, when TM3 cells were singly treated with BAPTA-AM, the release of intracellular Ca^2+^ was inhibited, which resulted in a block of physiological process, consequently led to apoptosis. It suggested that Ca^2+^ is necessary for cell survival, confirming Wang’s experimental results [32]. However, the increase of cytosolic free Ca^2+^ leads to changes in mitochondrial membrane potential and permeability transition, as well as cytochrome c release into the cytosol, resulting in the activation of the caspase cascade and consequent apoptosis [33]. As we observed in our experiment, T-2 toxin enhances the intracellular Ca^2+^ concentration and promotes the expression of cleaved caspase-3 in a time-dependent manner, but the effect is blocked in the presence of the general Ca^2+^ inhibitor BAPTA-AM. After co-treatment of T-2 toxin with BAPTA-AM, the T-2 toxin-mediated abnormal elevation of Ca^2+^ was attenuated by BAPTA-AM, and then the apoptosis was decreased. This indicated that T-2 toxin induced apoptosis in TM3 cells via the promotion of Ca^2+^ production. The potential mechanism may be that an overload of intracellular Ca^2+^ concentration caused energy dissipation via Ca^2+^ cycling [34], and it is likely to cause a decrease in the mitochondrial membrane potential [35].

mTORC1 (the mammalian target of rapamycin complex 1), formed by mTOR, Raptor, mLST8, PRAS40, and DEPTOR, regulates cell growth by promoting protein synthesis through phosphorylating the translational regulators S6 kinases (S6K1) and eukaryotic translation initiation factor 4E (4E-BP1) [36]. When compared with mTORC1, there is less knowledge of mTORC2. As reported, mTORC2 directly activates Akt, a kinase that regulates cellular processes, through the phosphorylation of its hydrophobic motif (Ser473) [37,38]. In our experiment, MHY1485 mitigated T-2 toxin-mediated suppression of phosphorylation of Akt at Ser473, verifying that the mTOR/Akt-473 pathway is involved in T-2 toxin-induced apoptosis. In several previous studies, the phosphorylation of mTOR was shown to depend on the concentration of intracellular Ca^2+^ [39,40]. However, in this study, mTOR activator MHY1485 attenuated the T-2 toxin-induced intracellular Ca^2+^ concentration, consequently protecting TM3 cells from apoptosis. It has reported that Ca^2+^ transport between endoplasmic reticulum and mitochondria, resulting the decrease of cellular adenosine triphosphate (ATP) and consequently activating autophagy via an mTOR-independent mechanism, as the study from Shin et al reported, mTORC1 inactivation contributes to the upregulation of autophagy [41]. Wu’s result shows that T-2 toxin treatment can induce both apoptosis and autophagy in L02 cells; however, the activation of autophagy has a potential protective process to against apoptosis induced by T-2 toxin [42]. In this study, T-2 toxin suppresses the both of mTORC1 and mTORC2, but there showed no protective effects of autophagy on T-2 toxin mediated apoptosis. The underlying mechanism is related to the duration of exposure and the concentration of T-2 toxin, which is worthy to further study.

## 4. Materials and Methods

### 4.1. Materials

T-2 toxin was purchased from BBI (Markham, ON, Canada). [4,5-Dimethyl-thiazol-2-yl]-2,5-diphenyltetrazolium bromide (MTT) was purchased from Sigma-Aldrich (Munich, Bavaria, Germany). Fetal bovine serum (FBS) was obtained from HyClone (Logan, UT, USA). Dulbecco’s modified eagle medium (DMEM)/nutrient mixture (Ham’s) F-12 with HEPES and horse serum was purchased from Gibco (Carlsbad, CA USA). The Annexin V-FITC apoptosis detection kit was purchased from Nanjing KeyGen Biotech. Co. Ltd. (Nanjing, China). The primary antibodies for p-mTOR ser473 and p-mTORser2448 were obtained from Abcam (Cambridge, Cambridge, UK). The primary antibodies of mTOR, p-AKTser473, AKT, caspase-3, and β-actin were obtained from Proteintech (Chicago, IL, USA), and the signal was visualized by using HRP-conjugated secondary antibodies that were obtained from Proteintech. BAPTA-AM and MHY1485 were purchased from ApexBio (Houston, TX, USA).

### 4.2. Cell Culture

TM3 Leydig cells were cultured and maintained in DMEM containing 10% fetal bovine serum (FBS) and 1% penicillin/streptavidin (Gibco-BRL, Grand Island, NY, USA) at 37 °C with 5% CO_2_. For each assay, cells were seeded onto culture dishes and routinely cultured for 24 h, and then treated with T-2 toxin for different times.

### 4.3. Drug Treatment

T-2 toxin was dissolved in 100% ethyl alcohol and further diluted with serum-free DMEM/F-12 to achieve the indicated final concentrate (100 nM) with vehicles as control (1% ethyl alcohol). BAPTA-AM, a highly selective and efficient Ca^2+^ chelator, and MHY1485, a potent, cell-permeable mTOR agonist, were treated on TM3 cells. TM3 Leydig cells was performed by pretreating the cells with BAPTA-AM (1 μM) or MHY1485 (10 μM) for 30 min and then continued with subsequent T-2 toxin (100 nM) treatment for 24 h.

### 4.4. Growth Inhibition Assay

Cell viability was evaluated with the MTT assay according to the manufacturer’s instructions. Briefly, 6 × 103 cells were seeded in 96-well plates, followed by overnight incubation. Serum-free media containing 100 nM T-2 toxin was then substituted for 10% FBS media. After 12–48 h, 20 µL of a 5 mg/mL solution of MTT was added, and then the cells were incubated for 4 h. Subsequently, after removing the supernatant, DMSO (150 µL) was added to dissolve the precipitate. The absorbance (optical density) of each well was measured at 492 nm using a microplate reader (Infinite^®^ M1000 Pro, Tecan, Salzburg, Austria). The data of the control treatment was considered to be 100% viable.

### 4.5. Western Blotting

TM3 cells were lysed in cold RIPA buffer (50 mM Tris–HCl pH 7.4, 150 mM NaCl, 0.25% sodium deoxycholate, 1% NP-40, 0.1% SDS, 10% glycerol) including protease inhibitors complex after treatment with T-2 toxin for predetermined times. Samples were separated in a 10% polyacrylamide gel and then transferred onto PVDF membranes (Immobilon-P Transfer membrane, Millipore, Billerica, MA, USA). The membranes were blocked with TBS-T buffer (20 mM Tris, 137 mM NaCl, pH 7.6, 0.1% Tween-20) containing 5% gelatin for 1 h at room temperature and subsequently probed at 4 °C overnight with special p-Akt, p-mTOR ser473, p-mTORser2448, Procaspase-3, cleaved caspase-3, or β-actin antibodies (Santa Cruz Biotechnology, Santa Cruz, CA, USA), and then incubated with goat anti-rabbit IgG or goat anti-mouse IgG as secondary antibodies (Santa Cruz Biotechnology, Santa Cruz, CA, USA) for 1 h at room temperature.

### 4.6. Detection of Apoptosis

TM3 cells were treated with 100 nM for 0, 12, 24, or 42 h. Afterward cells were digested by EDTA-free tryptic and centrifuged at 2000 rpm for 5–10 min. Precipitates were collected and washed with pre-cooled PBS. Cells were then stained with AnnexinV-FITC to quantify the percentage of cells undergoing apoptosis, and the necrotic cells were counterstained with propidium iodide (PI). Apoptosis was analyzed using flow cytometry (FACS Scan and CellQuest Pro software version 5.1; BD Biosciences, Franklin Lakes, NJ, USA).

### 4.7. Measurement of Intracellular Ca^2+^ Concentration

Changes in intracellular Ca^2+^concentration were assessed using the Ca^2+^-sensitive fluorescent indictor dye Fura 2-AM (Santa Crut Biotechnology, Inc.). Briefly, TM3 cells were washed with Ca^2+^-free buffer, including 120 mM NaCl, 5 mM KCl, 1 mM MgSO_4_, 0.96 mM NaH_2_PO_4_, 0.2% glucose, 0.1% bovine serum albumin (BSA), and 20 mM HEPES (pH = 7.2) and were loaded with 6 µM of Fura2-AM at 37 °C for 30 min. Fluorescence intensity was measured at excitation wavelengths of 340 and 380 nm. Images were acquired using an Olympus BX 51W1I upright microscope (Shinjuku-ku, Tokyo, Japan).

### 4.8. Statistical Analysis

Each experiment was performed and repeated at least three times. The results were analyzed using SPSS Statistics Base 19.0 (SPSS Inc., Chicago, IL, USA). *p* < 0.05 was considered to be significant. Values are expressed as the mean ± SD.

## 5. Conclusions

In conclusion, our results suggest that the inhibition of mTOR/Akt signaling pathway and the promotion of intracellular Ca^2+^ concentration are induction factors of apoptosis that is induced by T-2 toxin in TM3 cells. mTOR/Akt signaling pathway protects against apoptosis induced by T-2 toxin by decreasing the Ca^2+^ in TM3 cells. It proves that T-2 toxin induces apoptosis by inhibiting mTORC2/AKT to promote Ca^2+^ production in TM3 cells. The findings can provide a new insight for developing effective drugs and therapeutic targets to treat the damage by T-2 toxin.
